# 局限期小细胞肺癌同步推量调强放疗的Ⅰ期/Ⅱ期临床研究

**DOI:** 10.3779/j.issn.1009-3419.2017.01.04

**Published:** 2017-01-20

**Authors:** 静 尤, 会明 于, 马小薇 宋, 晨 石, 晓航 王, 晔 郑, 荣 余, 安辉 石, 广迎 朱

**Affiliations:** 100142 北京，北京大学肿瘤医院暨北京市肿瘤防治研究所，恶性肿瘤发病机制及转化研究教育部重点实验室，放疗科 Key Laboratory of Carcinogenesis and Translational Research (Ministry of Education), Department of Radiation Oncology, Peking University Cancer Hospital & Institute, Beijing 100142, China

**Keywords:** 肺肿瘤, 同步放化疗, 同步推量, Lung neoplasms, Concurrent chemo-radiotherapy, Simultaneous integrated boost

## Abstract

**背景与目的:**

同步放化疗是局限期小细胞肺癌的标准治疗，但患者局部复发率和远处转移率仍较高，本研究旨在评估同步推量调强放疗治疗局限期小细胞肺癌的安全性和有效性。

**方法:**

符合局限期小细胞肺癌的患者纳入研究行同步放化疗，放疗采用每日两次方案，应用经典“3+3”模式对肿瘤大体体积（gross target volume, GTV）进行同步推量剂量递增，设定为三个剂量梯度，分别为45 Gy/30 f（单次剂量1.50 Gy）、50 Gy/30 f（单次剂量1.67 Gy）和54 Gy/30 f（单次剂量1.80 Gy）。计划靶体积均为45 Gy/30 f。主要研究终点为放疗期间及结束3个月内毒性反应。次要研究终点包括1年生存率、无进展生存期、局部无进展生存期。

**结果:**

研究共入组26例患者，中位年龄为52岁（30岁-68岁）。26例患者中，1例出现3级放射性食管炎，未观察到3级及以上放射性肺炎。中位随访时间11.2（3.2-36.2）个月，1年生存率、无进展生存率和局部无进展生存率分别为89.0%、51.0%和85.0%。

**结论:**

局限期小细胞肺癌采用化疗联合同步推量调强放疗，将GTV由45 Gy提升至54 Gy是安全有效的。

## Abstract (en)

**Background and objective:**

Twice-daily radiation concurrent with chemotherapy is one of the standard methods for limited-stage small cell lung cancer. The study was to evaluate the feasibility of chemotherapy concurrent with dose-escalating twice-daily radiotherapy by simultaneous integrated boost intensity-modulated radiation therapy (SIB-IMRT) approach in patients with limited-stage small cell lung cancer.

**Methods:**

Patients with limited-stage small cell lung cancer were included, treated with twice-daily radiotherapy by SIB-IMRT concurrent with chemotherapy of etoposide plus cisplatin. Dose escalation was conducted by "classical" 3+3 methods with three patients enrolled in each dose level. The therapeutic gross tumor volume (GTV) was treated according to three consecutive dose levels *i*.*e*., 45 Gy at 1.5 Gy twice daily, 50 Gy at 1.67 Gy twice daily and 54 Gy at 1.8 Gy twice daily. The planning target volume (PTV) received a dose of 45 Gy delivered in 30 fractions of 1.5 Gy. The primary endpoints were acute toxicities. The secondary endpoints included overall survival (OS), progression-free survival (PFS) and loco-regional failure-free survival (LRFFS) at 1-year of follow-up.

**Results:**

Twenty men and six women were included. The median age was 52 (30-68) months. 12 patients experienced grade 2 acute esophagitis, and 1 patient developed grade 3 acute esophagitis. Only 3 patients developed Grade 2 pneumonitis. Grade 3 or higher radiation-related pneumonia was not observed. None died of treatment-related causes. With median follow-up of 11.2 months (3.2-36.2 months), 1-year OS, PFS and LRFFS were 89.0%, 51.0% and 85.0%, respectively.

**Conclusion:**

Dose escalation for twice-daily radiation concurrent with chemotherapy in LS-SCLC has been safely achieved up to 54 Gy for GTV using SIB-IMRT technique.

肺癌仍是全球发病率较高的一种恶性肿瘤。2015年，我国新诊断肺癌约733, 300例，死亡约610, 200例^[1]^。小细胞肺癌约占肺癌的15%^[2]^，其中，40%为局限期。同步放化疗是局限期小细胞肺癌（limited-stage small cell lung cancer, LS-SCLC）的主要治疗方式^[3, 4]^。由于小细胞肺癌增殖较快，一些研究尝试采用加速超分割的方式进行放疗。目前，每日两次的加速超分割放疗联合同步化疗是美国国立综合癌症网络（national comprehensive cancer network, NCCN）指南中LS-SCLC患者的Ⅰ类推荐。尽管小细胞肺癌对放化疗敏感，但由于其较高的局部失败率和远处转移率，往往预后较差。随着放疗技术的进展，近些年来，有不少研究探讨增加放疗剂量来提高局部控制率进而提高生存。本研究旨在评估同步推量调强放疗（simultaneous integrated boost-intensity modulated radiotherapy, SIB-IMRT）治疗LS-SCLC的安全性和有效性。

## 1 资料与方法

### 1.1 入组条件

经病理组织学或细胞学证实的小细胞肺癌患者；根据美国抗癌联盟会（American Joint Committee on Cancer, AJCC）第7版分期^[5]^为局限期（分期检查包括胸部增强电子计算机断层扫描（computed tomography, CT）、腹部超声/CT、颈部淋巴结超声、头核磁共振成像（magnetic resonance imaging, MRI）、骨扫描或头部增强MRI+全身PET/CT）；年龄18岁-70岁；东部肿瘤协作组（Eastern Cooperative Oncology Group, ECOG）评分0分-1分；无严重合并症；中性粒细胞绝对值≥2.0×10^9^/L，血红蛋白≥100 g/L，血小板≥100×10^9^/L；肌酐值≤1.5倍正常上限；总胆红素≤2.5倍正常上限；谷丙转氨酶≤2.5倍正常上限，谷草转氨酶≤2.5倍正常上限。排除标准：有远处转移；6个月内出现过心肌梗死；既往因其他疾病接受过放疗或化疗。所有患者入组前均签署知情同意书。

### 1.2 放化疗方案

患者化疗方案为依托泊苷+顺铂，共4个周期，每21天为1周期。具体为依托泊苷100 mg/m^2^，d1-d3，d22-d24，d43-d45，d64-d66；顺铂75 mg/m^2^，d1，d22，d43，d64。对于病灶较大的患者，可以先进行2周期诱导化疗后再进行同步放化疗。

### 1.3 放疗

所有患者均采用胸部增强CT进行定位扫描，扫描层厚为5 mm，扫描范围为下颌骨下缘至肝下缘。放疗采用瓦里安直线加速器，6 MV-10 MV X线。靶区勾画根据ICRU62号文件的定义。大体靶体积（gross target volume, GTV）定义为影像学可见的原发灶以及阳性淋巴结。原发灶临床靶体积（clinical target volume, CTV）为原发灶GTV基础上外括0.8 cm，再根据解剖边界进行调整；淋巴结CT为GTV基础上外扩0.8 cm，并参考化疗前的阳性淋巴结引流区。运动靶体积（internal target volume, ITV）为CTV基础上根据呼吸动度进行调整。计划靶体积（planning target volume, PTV）为ITV基础上外括0.5 cm。放疗采用每日两次方案，同一日两次放疗间隔时间≥6 h，5次/周。采用经典“3+3”模式对GTV进行同步推量剂量递增。GTV设定为三个剂量梯度：第一剂量梯度为45 Gy/30 f，单次剂量为1.50 Gy；第二剂量梯度为50 Gy/30 f，单次剂量1.67 Gy，第三剂量梯度为54 Gy/30 f，单次剂量为1.80 Gy。PTV均设定为45 Gy/30 f。剂量限制性毒性设定为出现3级或3级以上非血液学毒性。Ⅰ期研究中在第一剂量梯度若无患者出现3级或3级以上非血液学毒性，则直接进入下一剂量梯度；有1例出现3级或3级以上非血液学毒性，则继续纳入3例患者，若6例患者有2例出现3级或3级以上非血液学毒性，则上一剂量梯度作为最大耐受剂量；否则继续进入下一剂量梯度，以此类推。对于放化疗后评效达到完全缓解（complete response, CR）或部分缓解（partial response, PR）的患者，在治疗全部结束后4周进行脑预防性放疗，剂量为全脑25 Gy/10 f，1次/天，5次/周。此研究的流程图见图 1。

**1:**
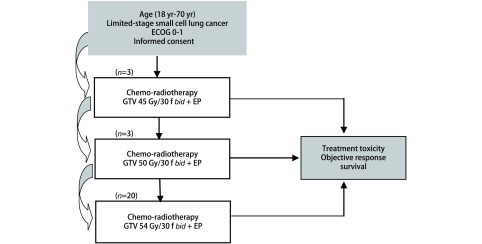
局限期小细胞肺癌行同步推量调强放疗联合化疗的流程图 Study workflow of concurrent chemotherapy with SIB-IMRT for all patients with limited-stage small cell lung cancer. SIB-IMRT: simultaneous integrated boost-intensity modulated radiation therapy.

### 1.4 疗效及毒性评价

肿瘤评效需结合胸部CT、腹部超声/CT、颈部淋巴结超声、头部增强MRI和全身骨扫描，根据实体瘤评效标准1.1版本（Response Evaluation Criteria in Solid Tumors, RECIST 1.1）^[6]^进行评效。放疗期间治疗相关毒性每周评价一次，根据国际癌症组织常见不良反应标准4.0（National Cancer Institute Common Terminology Criteria for Adverse Events, NCI-RECIST 4.0）标准^[7]^进行评价。

### 1.5 随访

放疗结束后前两年内每3个月随访1次，之后3年，每6个月随访1次，之后每年随访1次。内容包括体格检查、血常规、血生化、肿瘤标志物及影像学检查（包括胸部CT、腹部超声/CT、颈部淋巴结超声、头MRI和骨扫描，其中前5年全身骨扫描每6个月进行一次）。

### 1.6 统计学方法

所有数据资料采用SPSS 19.0软件进行处理。总生存期（overall survival, OS）定义为从诊断日期开始至任何原因所致死亡日期或末次随访日期。无进展生存期（progression-free survival, PFS）定义为从诊断日期开始至疾病进展或末次随访日期。局部无复发生存期（loco-regional failure-free survival, LRFFS）定义为从诊断日期开始至局部失败或末次随访日期。采用*Kaplan*-*Meier*法分析OS、PFS和LRFFS。

## 2 结果

### 2.1 患者临床特点

2012年12月-2016年5月，北京大学肿瘤医院放疗科共纳入26例LS-SCLC患者。患者具体临床特征包括：年龄、性别、ECOG评分、TNM分期、诱导化疗（表 1）。第一剂量梯度（GTV 45 Gy）入组3例患者，第二剂量梯度（GTV 50 Gy）入组3例患者，第一、第二剂量梯度未出现剂量限制性毒性；第三剂量梯度（GTV 54 Gy）入组20例患者。所有患者中，20例（76.9%）为男性，6例（23.1%）为女性。中位年龄为52岁（30岁-68岁）。42.3%为Ⅲa期，46.2%为Ⅲb期。20例患者（76.9%）接受过诱导化疗。

**1:** 局限期小细胞肺癌患者临床特征(*n*=26) Patient characteristics with limited-stage small cell lung cancer (LS-SCLC) (*n*=26)

Clinical Characteristics	Dose level			Total
	Dose level Ⅰ	Dose level Ⅱ	Dose level Ⅲ	
Number of patients	3	3	20	26
Age (yr) [Median (range)]	49 (47-56)	43 (43-51)	56.6 (30-68)	52 (30-68)
Gender [*n* (%)]				
Male	2 (66.7)	3 (100)	15 (75.0)	20 (76.9)
Female	1 (33.3)	0 (0)	5 (25.0)	6 (23.1)
ECOG performance [*n* (%)]				
0	2 (66.7)	2 (66.7)	13 (65.0)	17 (65.4)
1	1 (33.3)	1 (33.3)	7 (35.0)	9 (34.6)
T stage [*n* (%)]				
1	0 (0)	0 (0)	5 (25.0)	5 (19.2)
2	1 (33.3)	2 (66.7)	6 (30.0)	9 (34.6)
3	1 (33.3)	0 (0)	1 (5.0)	2 (7.7)
4	1 (33.3)	1 (33.3)	8 (40.0)	10 (38.5)
N stage [*n* (%)]				
0	0 (0)	0 (0)	1 (5.0)	1 (3.8)
1	0 (0)	1 (33.3)	3 (15.0)	4 (15.4)
2	2 (66.7)	2 (66.7)	11 (55.0)	15 (57.7)
3	1 (33.3)	0 (0)	5 (25.0)	6 (23.1)
AJCC stage [*n* (%)]				
Ⅱa	0 (0)	0 (0)	3 (15.0)	3 (11.5)
Ⅱb	0 (0)	0 (0)	0 (0)	0 (0)
Ⅲa	2 (66.7)	1 (33.3)	8 (40.0)	11 (42.3)
Ⅲb	1 (33.3)	2 (66.7)	9 (45.0)	12 (46.2)
Induction chemotherapy [*n* (%)]				
Yes	3 (100)	1 (33.3)	16 (80.0)	20 (76.9)
No	0 (0)	2 (66.7)	4 (20.0)	6 (23.1)
Prophylactic cranial irradiation [*n* (%)]				
Yes	2 (66.7)	1 (33.3)	15 (75.0)	18 (69.2)
No	1 (33.3)	2 (66.7)	5 (25.0)	8 (30.8)
EOCG: Eastern Cooperative Oncology Group; AJCC: American Joint Committee on Cancer.				

### 2.2 安全性和毒性

所有患者均完成了治疗计划。治疗相关毒性见表 2。血液学毒性方面，13例患者（50.0%）出现3度中性粒细胞减少，4例患者（15.4%）出现4度中性粒细胞减少。1例患者（3.8%）出现3度贫血，3例患者（11.5%）出现3度血小板减少。无患者出现4度贫血和血小板减少。非血液学毒性方面，第一剂量水平组（共3例）和第2剂量水平组（共3例）未出现3度非血液学毒性，第三剂量水平组（共20例）有1例患者出现3度放射性食管炎。无患者出现3度或3度以上放射性肺炎。无患者因治疗相关毒性导致死亡。

**2:** 局限期小细胞肺癌患者治疗相关毒性(*n*=26) [*n* (%)] Treatment-related toxicities of patients with LS-SCLC (*n*=26) [*n* (%)]

	Grade 0	Grade 1	Grade 2	Grade 3	Grade 4	Grade 5
Hematological toxicity						
Leukemia	0 (0)	0 (0)	10 (38.5)	15 (57.7)	1 (3.8)	-
Neutropenia	1 (3.8)	1 (3.8)	7 (26.9)	13 (50.0)	4 (15.4)	-
Anemia	6 (23.1)	14 (53.8)	5 (19.2)	1 (3.8)	0 (0)	-
Thrombocytopenia	9 (34.6)	6 (23.1)	8 (30.8)	3 (11.5)	0 (0)	-
Non-hematological toxicity						
Fatigue	2 (7.7)	17 (65.4)	6 (23.1)	1 (3.8)	0 (0)	0 (0)
Nausea	16 (61.5)	8 (30.8)	2 (7.7)	0 (0)	0 (0)	0 (0)
Anorexia	21 (80.8)	3 (11.5)	2 (7.7)	0 (0)	0 (0)	0 (0)
Dysphagia	15 (57.7)	2 (7.7)	8 (30.8)	1 (3.8)	0 (0)	0 (0)
Esophagitis	12 (46.2)	1 (3.8)	12 (46.2)	1 (3.8)	0 (0)	0 (0)
Pneumonitis	21 (80.0)	2 (7.7)	3 (11.5)	0 (0)	0 (0)	0 (0)
Hepatic injury	20 (76.9)	4 (15.4)	1 (3.8)	1 (3.8)	0 (0)	0 (0)
hyperbilirubinemia	22 (84.6)	3 (11.5)	1 (3.8)	0 (0)	0 (0)	0 (0)

### 2.3 疗效评估

接受诱导化疗的20例患者化疗结束后，16例患者达到PR，4例评效稳定（stable disease, SD），无患者达到CR。所有治疗完成后进行评效，11例患者（42.3%）达到CR，13例患者（50%）达到PR，2例患者（7.7%）出现病情进展。总客观反应率为（CR+PR）为92.3%。

### 2.4 生存

中位随访时间为11.2（3.2-36.2）个月，中位OS和PFS尚未达到。所有患者1年OS、PFS和LRFFS分别为89.0%、51.0%和85.0%（图 2-图 4）。至末次随访时，26例患者中，19例患者（73.1%）仍然存活，14例患者（53.8%）未出现疾病进展。有3例患者（11.5%）出现局部复发，9例患者（34.6%）出现远处转移。这9例患者中，5例（55.6%）出现脑转移，3例（33.3%）出现肝转移，1例（11.1%）出现骨转移。其中，5例脑转移患者中有4例未接受脑预防性放疗（prophylactic cranial irradiation, PCI）（2例在治疗结束时即出现脑转移，2例患者拒绝PCI，后期随访中出现脑转移）。

**2:**
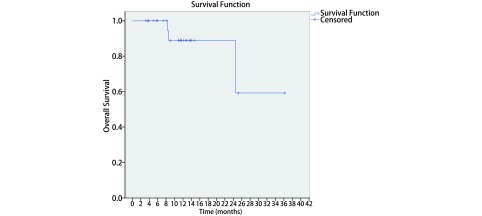
局限期小细胞肺癌患者行同步推量调强放疗联合化疗的总生存 The overall survival of concurrent chemotherapy with SIB-IMRT for all patients with LS-SCLC

**3:**
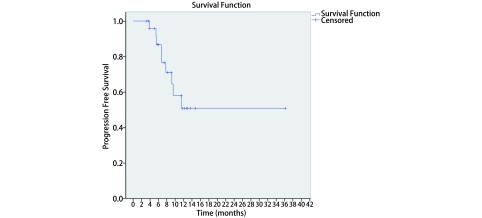
局限期小细胞肺癌患者行同步推量调强放疗联合化疗的无进展生存 The progression-free survival of concurrent chemotherapy with SIB-IMRT for all patients with LS-SCLC

**4:**
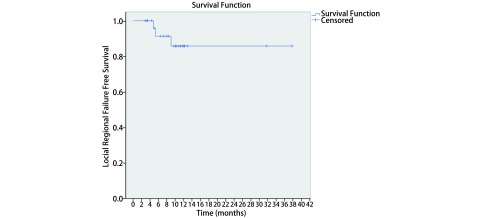
局限期小细胞肺癌患者行同步推量调强放疗联合化疗的局部无复发生存 The Local Regional Failure-free Survival of concurrent chemotherapy with SIB-IMRT for all patients with LS-SCLC

## 3 讨论

目前，同步放化疗是LS-SCLC的标准治疗，其中，NCCN指南推荐进行加速超分割放疗或常规分割放疗。常规分割放疗的标准剂量为60 Gy-70 Gy，分30次-35次完成，单次剂量为2 Gy；加速超分割放疗的推荐剂量为45 Gy，分30次完成，单次剂量为1.5 Gy。其中，每日2次的加速超分割放疗模式是基于INT 0096研究^[8]^，研究对比了LS-SCLC患者行加速超分割放疗和常规分割放疗的优劣。该研究入组了417例LS-SCLC患者，随机分为两组，两组放疗剂量均为45 Gy，分30次完成。结果显示，加速超分割放疗5年生存率优于常规分割放疗（26% *vs* 16%）；局部控制率方面，加速超分割放疗使局部失败率由52%降至36%。

为了进一步提高局部控制率，进而提高生存，更多的研究探讨了通过提高胸部放疗剂量联合化疗治疗LS-SCLC的治疗模式^[9, 10]^。RTOG 0239研究^[11]^纳入了71例LS-SCLC患者，通过加速超分割放疗结合常规放疗将胸部放疗剂量提升至61.2 Gy（第1天-第22天行常规放疗，单次剂量1.8 Gy；第23天-第33天行每日两次放疗，单次剂量1.8 Gy），同步EP方案化疗，2年OS和局部控制率分别为36.0%和73.0%。Han等^[12]^通过采用序贯放化疗治疗LS-SCLC，其中放疗采用SIB-IMRT进行加速超分割：GTV剂量为57 Gy（单次剂量1.9 Gy），CTV剂量为51 Gy（单次剂量1.7 Gy），PTV剂量为45 Gy（单次剂量1.5 Gy）。结果显示，2年OS和PFS分别为68.5%和40.7%。而我们这项研究对LS-SCLC进行同步放化疗，评估了应用SIB-IMRT的有效性和安全性，得到了较好的生存结果，毒性可耐受。

既往研究显示，加速超分割较常规放疗所致食管炎发生率增加，从而限制了放疗剂量的提高^[13]^，而本项研究在加速超分割的基础上通过SIB-IMRT进行GTV剂量递增，治疗相关毒性耐受性良好。所有患者出现1级、2级和3级放射性食管炎的发生率分别为7.6%（2例）、30.8%（8例）和3.8%（1例），较其他研究^[8, 14]^中放射性食管炎发生率要低。放射性肺炎方面，仅有3例（11.5%）患者出现2级放射性肺炎，未观察到3级及以上放射性肺炎发生，也明显低于其他研究中放射性肺炎发生率^[12, 15, 16]^。

此研究中放疗相关毒性明显低于其他研究，分析主要有以下三个原因。首先，本研究应用调强放疗技术，与既往的三维适形放疗技术（3-dimensional conformal radiation therapy, 3D-CRT）比较，能够更好的提高肿瘤区域的放疗剂量同时减少正常组织的照射剂量。其次，采用SIB-IMRT进行剂量递增，使原发灶的放疗剂量增加以提高局部控制率，同时对亚临床病灶给予相对降低的剂量，降低了治疗相关不良反应的发生率。这项研究中危及器官限量包括双肺、心脏、食管，与其他研究^[12, 16, 17]^比较均较低，见表 3。近期一项*meta*分析显示，肺V20每增加1%，有症状的放射性肺炎发生率增加3%^[18]^。此研究中双肺V20平均值为24.2%，有症状的放射性肺炎发生率相应较低。此外，对于病灶较大的患者，本研究采用2周期诱导化疗来缩小肿瘤体积，有助于进一步降低放射性肺炎的发生^[19]^。

**3:** 局限期小细胞肺癌患者的危及器官限量(*n*=26) Dose volume parameter of organ at risk for patients with LS-SCLC (*n*=26)

Item	Data
GTV volume	
Median	68.5
Range	9.7-369.7
PTV volume	
Median	379.1
Range	124.1-1190.9
Total Lungs (Mean±SD)	
V20 (%)	24.2±0.9
V10 (%)	39.7±1.4
V5 (%)	55.5±1.8
MLD (cGy)	1364.4±42.4
Esophagus (Mean±SD)	
Dmean (cGy)	2757.4±159.0
Dmax (cGy)	5592.1±137.6
Heart (Mean±SD)	
V40 (%)	14.2±2.5
V45 (%)	7.3±2.1
Dmax (cGy)	1543.9±147.8
OAR: organs at risk; MLD: mean lung dose; V20: percentage of volume receiving≥20 Gy; V10: percentage of volume receiving≥10 Gy; V5: percentage of volume receiving≥5 Gy; Dmax: maximum dose; Dmean: mean dose; V40: percentage of volume receiving≥40 Gy; V45: percentage of volume receiving≥45 Gy.	

所有患者均根据治疗方案完成了治疗计划，毒性可耐受。通过SIB-IMRT技术，治疗总反应率达到92.3%。1年OS、PFS和LRFFS仍较高，分别为89.0%、51.0%和85.0%。但此研究存在样本量较小，随访时间较短的不足之处。对比常规分割放疗方案，每日两次放疗也存在一些优势。首先，每日两次放疗缩短了总的治疗时间，有利于提高LS-SCLC的局部控制率。其次，有研究显示，较早进行PCI能够降低脑转移发生率^[20]^。临床实践中也发现，LS-SCLC患者进行每日一次放疗结束后评效已出现脑转移，而每日两次放疗缩短了胸部放疗的时间，使患者尽早接受PCI治疗，从而有可能进一步降低脑转移发生率。此研究5例出现脑转移的患者中，有4例患者未接受PCI，其中，1例患者治疗结束时即出现脑转移，3例患者拒绝行PCI，后期随访中出现脑转移，也进一步强调了尽早进行PCI的重要性。

综上所述，LS-SCLC患者采用SIB-IMRT进行GTV剂量递增加速超分割放疗联合EP方案化疗是安全有效的。其有效性需要更大的样本量和随访时间以及前瞻性Ⅲ期随机对照研究来证实。
